# Multi-site phosphorylation regulates NeuroD4 activity during primary neurogenesis: a conserved mechanism amongst proneural proteins

**DOI:** 10.1186/s13064-015-0044-8

**Published:** 2015-06-18

**Authors:** Laura J. A. Hardwick, Anna Philpott

**Affiliations:** Department of Oncology, University of Cambridge, Hutchison/MRC Research Centre, Cambridge Biomedical Campus, Cambridge, CB2 0XZ UK

**Keywords:** NeuroD4, Neurogenesis, Phosphorylation, Proneural, bHLH, *Xenopus*

## Abstract

**Background:**

Basic Helix Loop Helix (bHLH) proneural transcription factors are master regulators of neurogenesis that act at multiple stages in this process. We have previously demonstrated that multi-site phosphorylation of two members of the proneural protein family, Ngn2 and Ascl1, limits their ability to drive neuronal differentiation when cyclin-dependent kinase levels are high, as would be found in rapidly cycling cells. Here we investigate potential phospho-regulation of proneural protein NeuroD4 (also known as Xath3), the *Xenopus* homologue of Math3/NeuroM, that functions downstream of Ngn2 in the neurogenic cascade.

**Results:**

Using the developing *Xenopus* embryo system, we show that NeuroD4 is expressed and phosphorylated during primary neurogenesis, and this phosphorylation limits its ability to drive neuronal differentiation. Phosphorylation of up to six serine/threonine-proline sites contributes additively to regulation of NeuroD4 proneural activity without altering neuronal subtype specification, and number rather than location of available phospho-sites is the key for limiting NeuroD4 activity. Mechanistically, a phospho-mutant NeuroD4 displays increased protein stability and enhanced chromatin binding relative to wild-type NeuroD4, resulting in transcriptional up-regulation of a range of target genes that further promote neuronal differentiation.

**Conclusions:**

Multi-site phosphorylation on serine/threonine-proline pairs is a widely conserved mechanism of limiting proneural protein activity, where it is the number of phosphorylated sites, rather than their location that determines protein activity. Hence, multi-site phosphorylation is very well suited to allow co-ordination of proneural protein activity with the cellular proline-directed kinase environment.

**Electronic supplementary material:**

The online version of this article (doi:10.1186/s13064-015-0044-8) contains supplementary material, which is available to authorized users.

## Background

Basic helix-loop-helix (bHLH) transcription factors are master regulators during development, acting in all three embryonic germ layers and establishing a complex network of interactions that direct cell fate towards specified lineages [1–4]. Within this family, proneural bHLH proteins such as Neurogenin2 (Ngn2) and Ascl1 have well-established roles as neuronal determination and differentiation factors [1, 5]; this function is highly conserved from invertebrate *Drosophila* [6] and through vertebrate models such as zebrafish and *Xenopus* [7], chick [8], rodent and humans [9]. A dynamic interplay exists whereby these proneural bHLH proteins on one hand cell autonomously drive differentiation, yet also non-cell autonomously promote progenitor maintenance through Notch signalling and the actions of inhibitory neurogenic bHLH proteins of the Hes family [10]. Moreover, their role in co-ordinating cell cycle and differentiation is also becoming apparent [11–13].

Proneural protein activity may lie at the heart of mechanisms that allow cell cycle exit to be co-ordinated with differentiation. For instance, we have previously demonstrated that both Ngn2 and Ascl1 undergo multi-site phosphorylation on serine-proline sites, and this directly reduces their ability to drive neuronal differentiation under conditions of high cyclin dependent kinase (cdk) activity [14–16]. Accordingly, phospho-mutant versions of Ngn2 and Ascl1 show enhanced proneural activity in both *Xenopus* embryos and mammalian cell models [14, 16]. This raises important questions: Firstly, is this mechanism of phospho-regulation conserved more widely amongst proneural bHLH transcription factors? Secondly, if phospho-regulation can influence generic proneural activity, does it also influence the subtype specification of neuronal progeny?

Here, we investigate phospho-regulation of the proneural protein NeuroD4 [17], the *Xenopus* protein also known as Xath3, and homologue of mammalian Math3/NeuroM. Focussing on *Xenopus* primary neurogenesis, we show that NeuroD4 is expressed and phosphorylated at neural plate stages in the developing embryo, and this limits its ability to drive primary neurogenesis. Phosphorylation of up to six sites additively contributes to regulation of NeuroD4 proneural activity, without altering neuronal subtype specification. Mechanistically, we demonstrate that phospho-mutant NeuroD4 has both enhanced protein stability relative to wild-type NeuroD4 *in vivo*, and also increased chromatin binding at neural plate stages. Our findings indicate that cell-cycle mediated multi-site phosphorylation is a widely conserved mechanism of regulating proneural protein activity.

## Results

### NeuroD4 contains conserved proline-directed phosphorylation sites

**Fig. 1:**
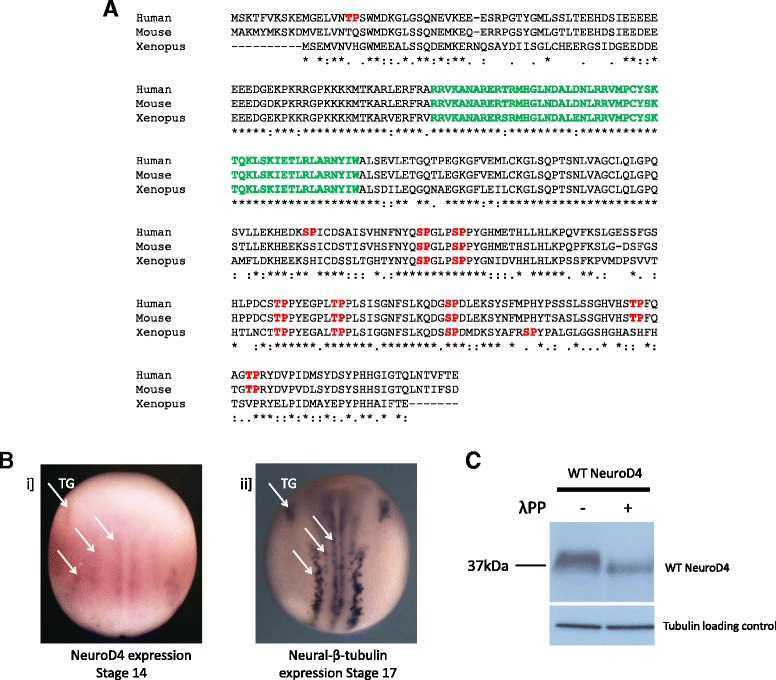
NeuroD4 is expressed and phosphorylated during primary neurogenesis (A-C). **a** Protein sequence alignment for human, mouse and *Xenopus* NeuroD4 homologues using Clustal W. The conserved bHLH domain is indicated in green and SP/TP sites are highlighted in red. A consensus line is also shown below the alignment to indicate the degree of conservation of amino acids at each position: (*) denotes identical residues in all three sequences; (:) denotes highly conserved residues; (.) denotes weakly conserved residues. **b** Endogenous expression of NeuroD4 at stage 14 (i) and neural-β-tubulin at stage 17 (ii) was determined by whole mount ISH (white arrows correspond to zones of primary neurogenesis; TG = trigeminal ganglia). Dorso-ventral views, anterior up, stages as indicated. **c** Western Blot analysis of extracts from stage 13 embryos injected with an HA-tagged wild type (WT) NeuroD4 mRNA, either with or without protein phosphatase treatment. Tubulin was used as a loading control

bHLH proteins are subdivided into families on the basis of sequence conservation, and *NeuroD4* is grouped in the Atonal family, related to the *Drosophila* proneural gene *atonal* [18]. Species homologues of each proneural protein often show a high degree of sequence conservation in the bHLH domain that mediates DNA-binding and protein dimerisation, but N and C terminal domains can be significantly divergent in sequence [1]. hese regions are suggested to be unstructured domains and present prime sites for post translational modifications such as phosphorylation [19]. Protein sequence alignment for human (genbank accession number NP 067014.2), mouse (genbank accession number NP 031527.1) and *Xenopus* (genbank accession number NP 001081213.1) NeuroD4 homologues show not only the strong sequence conservation in the bHLH domain, but also sequence conservation in potential proline-directed kinase sites (serine or threonine followed by a proline, Fig. 1a) that conform to the basic consensus sites for phosphorylation by cyclin dependent kinases (cdks) [20]. In NeuroD4 there are four serine-proline (SP) sites and two threonine-proline (TP) sites; five out of six of these sites are highly conserved with the mouse and human proteins, while the mouse and human proteins have additional non-conserved TP sites in the C terminus that are not shared with the *Xenopus* protein (Fig. 1a).

Limited evidence for phospho-regulation of NeuroD4 has been previously described; a phospho-mimetic version of NeuroD4 with aspartic acid replacing serine-89 within the basic domain impairs the expression of anterior neural markers without affecting the level of induced neurogenesis relative to wild-type (WT) NeuroD4 [17]. We set out to investigate a potential regulatory role of the SP/TP sites in NeuroD4 by exploring primary neurogenesis in the developing *Xenopus* embryo as a model system.

### NeuroD4 is expressed in the neural plate and phosphorylated during primary neurogenesis

The murine homologue Math3 is expressed in the developing spinal cord from embryonic day (E) 8.5 and then later in the developing brain and retina [17]. Similarly, expression of chick homologue NeuroM in spinal cord and optic tectum coincides with cells in a transition stage between proliferation in the ventricular zone (VZ) and differentiation and migration of post-mitotic neurons [21]. Consistent with playing a similar role in *Xenopus,* NeuroD4 expression is first detected from stage 12 in the presumptive neural plate [17], becoming localised at stage 14 to the trigeminal ganglia and three bilateral stripes of differentiating primary neurons (Fig. 1b); these give rise to somato-sensory (Rohon-Beard neurons), interneurons, and somato-motor neurons respectively [22]. NeuroD4 expression persists at a high level in the brain and retina [17]. Firstly, we investigated whether NeuroD4 is phosphorylated during primary neurogenesis in developing *Xenopus* embryos. Extracts were prepared from stage 13 embryos injected with an HA-tagged wild type (WT) NeuroD4 mRNA, and western blot analysis shows the presence of a broad band of NeuroD4 protein (Fig. 1c). Protein migration is significantly enhanced by treatment with a broad-spectrum phosphatase, demonstrating that NeuroD4 is phosphorylated on at least one site. We next investigated the effect of mutating potential SP and TP phosphorylation sites on NeuroD4 neurogenic function.

### A phospho-mutant NeuroD4 promotes enhanced neuronal differentiation *in vivo*

**Fig. 2:**
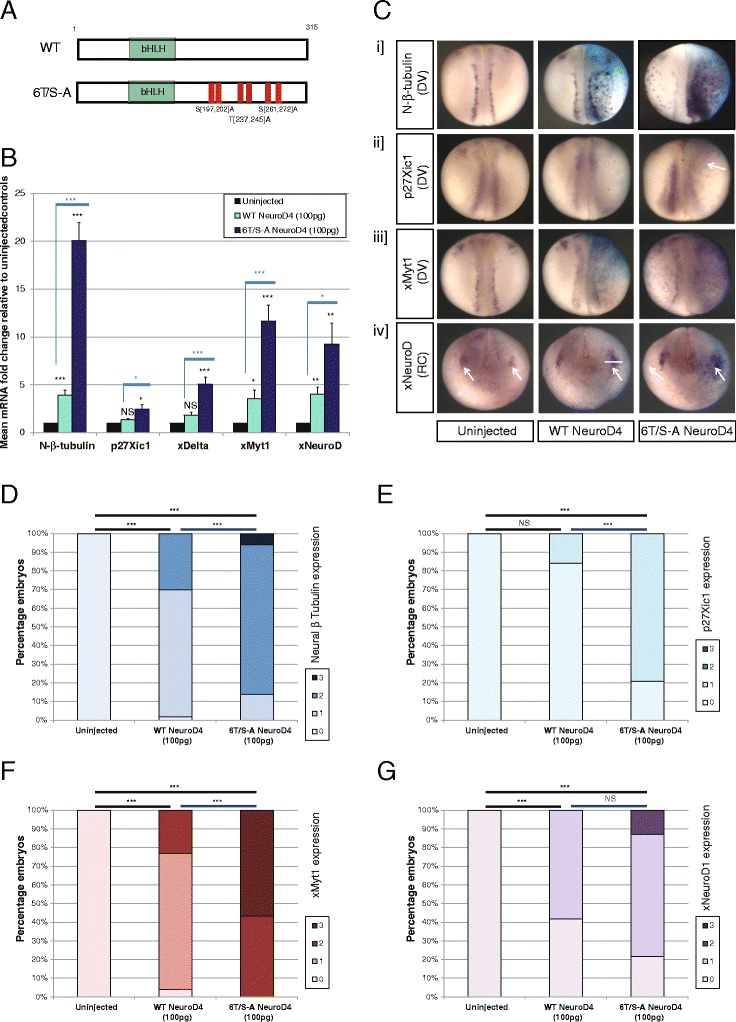
Phospho-mutant 6T/S-A NeuroD4 has increased proneural activity relative to WT NeuroD4 (**a**-**g**). **a** Schematic representation of WT NeuroD4 and full phospho-mutant 6T/S-A NeuroD4 protein sequences, indicating the relative positions of the six SP or TP sites that have been mutated to AP sites. **b**-**g** Two-cell stage embryos were unilaterally injected with 100 pg of either WT or 6T/S-A NeuroD4 mRNA, and at stage 18, gene expression was assayed by qRT-PCR (**b**), or by whole mount ISH (**d**-**g**) with representative embryo images shown in (**c**). For qRT-PCR analysis (**b**) significance is calculated as described in the methods section for phospho-mutant NeuroD4 relative to WT NeuroD4 (blue adjoining lines and stars) and relative to uninjected control embryos (shown with black stars) [*n* = 5]; (*p* < 0.05) = *; (*p* < 0.025) = **; (*p* < 0.0125) = ***. For ISH analysis, embryos were scored according to the scale described in Additional file 1 for expression of neural-β-tubulin (D [*n* = 100–115]), p27Xic1 (E [*n* = 24-33]), xMyt1 (F [*n* = 26-32]), and xNeuroD1 (G [*n* = 23-28]). Views in (C [i-iii]) are dorso-ventral (DV); views in (C [iv]) are rostro-caudal (RC) with dorsal surface facing up, trigeminal ganglia indicated by arrows. All images show injected side to the right

To determine the activity of NeuroD4 when phosphorylation on SP and TP sites is prevented, we generated a phospho-mutant version where all six SP/TP sites are mutated to alanine-proline (AP), creating 6T/S-A NeuroD4 (Fig. 2a). In order to compare their proneural activity, mRNA encoding WT and 6T/S-A NeuroD4 proteins were injected unilaterally into two-cell stage *Xenopus* embryos, and neural-β-tubulin expression was assayed at stage 18 (Fig. 2b-d). Consistent with previous reports [17, 23], over-expression of WT NeuroD4 produces a mild to moderate increase in neural-β-tubulin expression, seen in both the neural plate and lateral ectoderm of embryos. 6T/S-A NeuroD4 induces significantly greater ectopic neural-β-tubulin expression than that induced by WT NeuroD4, as judged by in situ hybridisation (ISH) and by qRT-PCR. Hence, preventing modification of SP/TP sites substantially enhances the proneural activity of NeuroD4, similar to our previous findings with Ngn2 and Ascl1 [14–16]. Phosphorylation on multiple SP and TP sites may therefore be a widely conserved mechanism for limiting the activity of bHLH proneural proteins.

### Enhanced target gene expression by phospho-mutant NeuroD4

Preventing multi-site phosphorylation of Ngn2 and Ascl1 enhances the transcriptional activation of a number of downstream targets. However, not all targets show a similar level of up-regulation by the phospho-mutant proteins compared to their wild-type counterparts [15, 16]. For instance xDelta, which is known to have an epigenetically “available” promoter shows similar up-regulation by WT and S-A mutant versions of Ngn2, while xNeuroD1 that requires more chromatin modification for activation shows substantially greater activation by the phospho-mutant compared to the wild-type protein [15, 24]. Hence epigenetic availability of target genes appears to contribute to the sensitivity of targets to Ngn2 phosphorylation. To investigate whether distinct downstream targets of NeuroD4 respond differently to NeuroD4 phospho-status, we investigated expression of xDelta [25] and xNeuroD1 [26] as markers of progenitor maintenance and neuronal differentiation respectively, along with other potential targets of NeuroD4 that can promote neuronal differentiation, namely xMyt1 [27] and p27Xic1 [28] (Fig. 2b-g).

WT NeuroD4 over-expression results in a four-fold increase in both xMyt1 and xNeuroD1 expression, but does not significantly up-regulate p27Xic1 or xDelta at this level of overexpressed mRNA. In contrast, 6T/S-A NeuroD4 up-regulates all four transcripts relative to both uninjected controls and WT NeuroD4 (Fig. 2b). Of note is the five-fold increase in xDelta expression by 6T/S-A NeuroD4 compared a negligible increase by WT NeuroD4; WT and 9S-A Ngn2 give similar up-regulation of xDelta [15], indicating differences in promoter activation by these two proneural proteins. In addition, 9S-A Ngn2 induces a fifty-fold increase in xNeuroD1 [15], while 6T/S-A NeuroD4 induces a much more modest nine-fold increase that appears largely localised to the trigeminal ganglia (Fig. 2c). This indicates that NeuroD4 may have additional requirements for regionally restricted co-factors to drive neuronal differentiation not needed by Ngn2. Thus, while xDelta and xNeuroD1 are downstream target for both Ngn2 and NeuroD4 [23], the dynamics of downstream promoter activation and their sensitivity to proneural protein phospho-status clearly differ between the two proneural proteins.

We have previously demonstrated that the cdk inhibitor (cdki) p27Xic1 is required for primary neurogenesis, playing a dual role in slowing the cell cycle and independently promoting differentiation by stabilisation of the Ngn2 protein [28]. However, perhaps surprisingly, p27Xic1 expression has not been shown to be up-regulated by overexpressed Ngn2 [29]. As NeuroD4 is expressed at the point of cell cycle exit, we explored whether NeuroD4 could transcriptionally activate p27Xic1, facilitating the transition from progenitor to differentiating neuron. WT NeuroD4 is unable to induce p27Xic1 expression, but 6T/S-A NeuroD4 induces a 2.5 fold increase in p27Xic1 message by qRT-PCR (Fig. 2b). Interestingly, in situ hybridisation reveals that p27Xic1 is induced by 6T/S-A NeuroD4 in the lateral ectoderm of the embryo but is also suppressed in the underlying myotome (Fig. 2c). This may potentially result in an underestimation of p27Xic1 induction by 6T/S-A NeuroD4 in the neural plate and ectoderm as measured by qRT-PCR, which nevertheless shows more than a 2-fold increase compared to uninjected embryos.

In summary, a phospho-mutant form of NeuroD4 displays substantially enhanced ability to drive neuronal differentiation in *Xenopus,* and in contrast to phospho-mutant Ngn2 [15], shows an enhanced expression of all the potential downstream targets tested, as compared to wild-type NeuroD4.

### NeuroD4 activity is limited by the number rather than the location of available phospho-sites

**Fig. 3:**
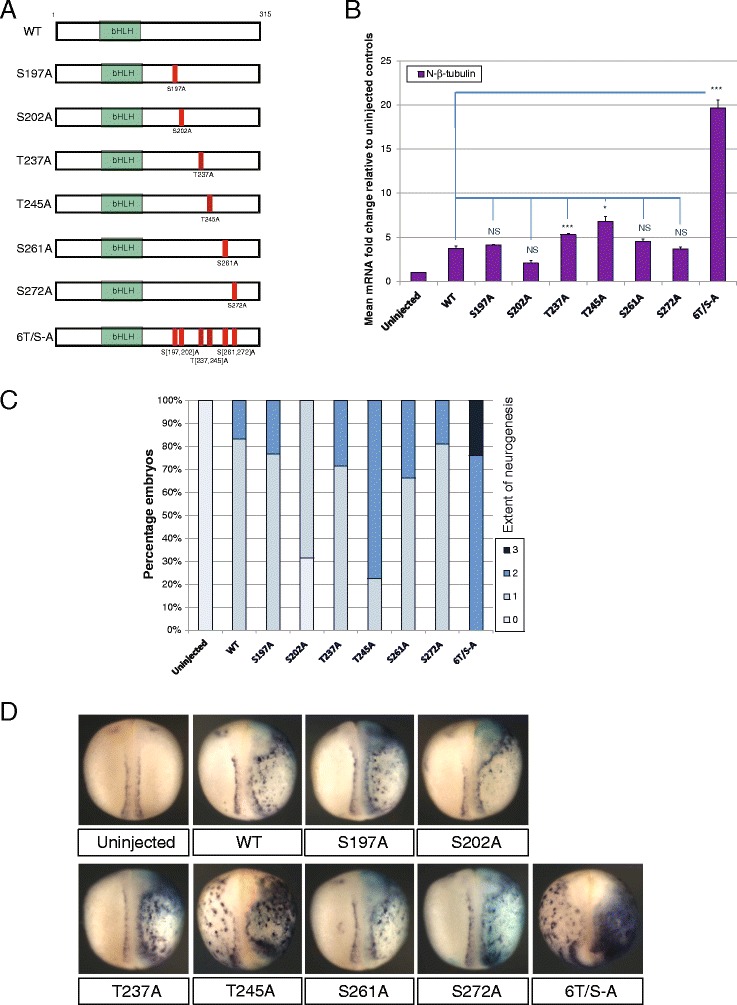
Similar levels of proneural activity are seen amongst single site mutants (**a**-**d**). **a** Schematic representation of the single site phospho-mutant NeuroD4 constructs demonstrating the relative position of the SP or TP site mutated to AP in each. Two-cell stage embryos were unilaterally injected with 100 pg mRNA of the respective NeuroD4 construct and assayed at stage 18 for expression of neural-β-tubulin by qRT-PCR (B[*n* = 3]), or whole embryo ISH (C[*n* = 51-77]) with representative images shown in (**d**). Views are dorso-ventral with injected side to the right; (*p* < 0.05) = *; (*p* < 0.025) = **; (*p* < 0.0125) = ***

In some bHLH proteins where phosphorylation of SP and/or TP sites has been shown to regulate protein activity, individual phosphorylation sites have particularly important regulatory significance, for example S200 in MyoD [30] or S147 in Olig2 [31]. In contrast, our earlier work indicates that it is the number of available phospho-sites in Ngn2, rather than their precise location, that controls Ngn2 differentiation activity [14, 15]. To investigate whether particular SP/TP sites have specific regulatory roles or whether it is the number of sites rather than their location that determines NeuroD4 activity, we generated a series of mutant versions of NeuroD4, starting with a panel in which individual SPs or TPs were mutated to AP (Fig. 3a), and tested their ability to promote ectopic neurogenesis after mRNA injection.

Mutation of individual SP sites generates mutants with activity similar to WT NeuroD4, while mutation of individual TP sites results in a small increase in activity (Fig. 3b-d). However, this increase is modest compared to the 20-fold enhancement seen with 6T/S-A NeuroD4, demonstrating that multiple SP/TP sites must be mutated to achieve maximal activity. SP and TP sites in NeuroD4 are found in pairs, and two of these pairs are found in highly conserved regions. It is possible that, when one of these sites is mutated, phosphorylation switches to its neighbour, but nevertheless phosphorylation at the location of these paired sites could be important for controlling NeuroD4 activity. Alternatively, the location of phosphorylation could be less important for regulation than the number of sites available.

**Fig. 4:**
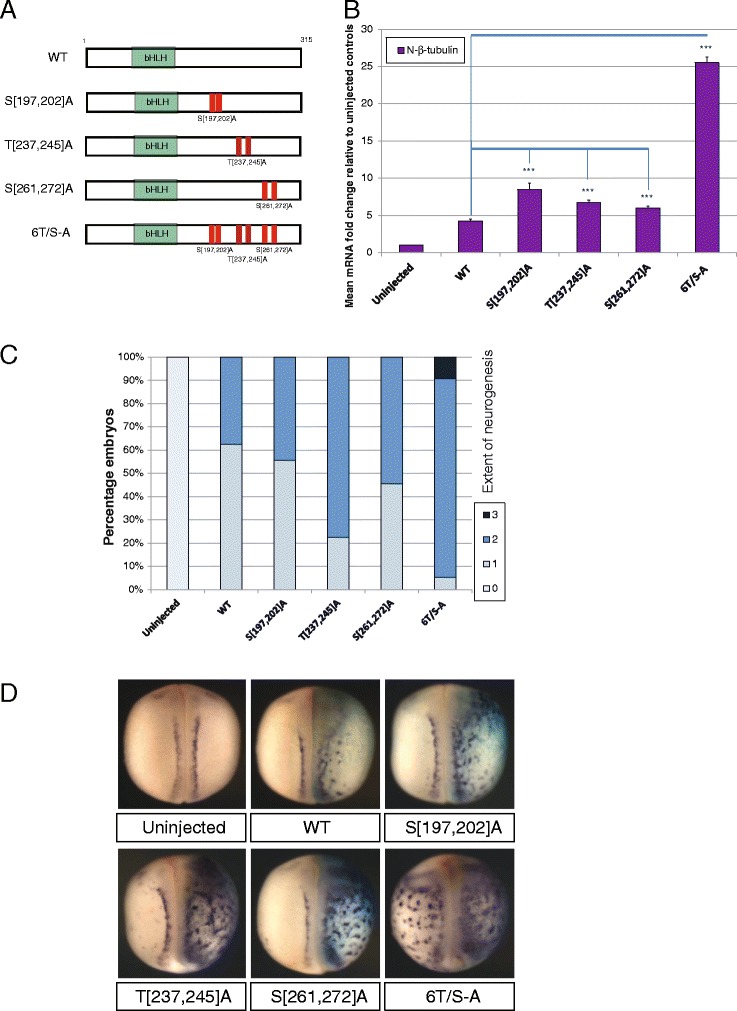
Similar levels of proneural activity are seen amongst paired site mutants (**a-d**). **a** Schematic representation of the paired site phospho-mutant NeuroD4 constructs. Two-cell stage embryos were unilaterally injected with 100 pg mRNA of the respective NeuroD4 construct and assayed at stage 18 for expression of neural-β-tubulin by qRT-PCR (B[*n* = 3]), or whole embryo ISH (C[*n* = 62-79]) with representative images shown in (**d**). Views are dorso-ventral with injected side to the right; (*p* < 0.05) = *; (*p* < 0.025) = **; (*p* < 0.0125) = ***

To distinguish between these alternative possibilities, a second panel of phospho-mutant constructs were made in which pairs of adjacent SP or TP sites were mutated together (Fig. 4a) and assayed in *Xenopus* embryos as described above. All three paired mutants produce a similar six to seven fold increase in neural-β-tubulin expression that is significantly higher than that induced by WT NeuroD4, but still substantially lower than that induced by the full phospho-mutant 6T/S-A NeuroD4 (Fig. 4b-e). Therefore, mutation of paired residues enhances NeuroD4 proneural activity compared to single site mutations, but the location of these paired sites does not influence the level of NeuroD4 activity; collective mutation of all six sites is required for the full neurogenic activity.

**Fig. 5:**
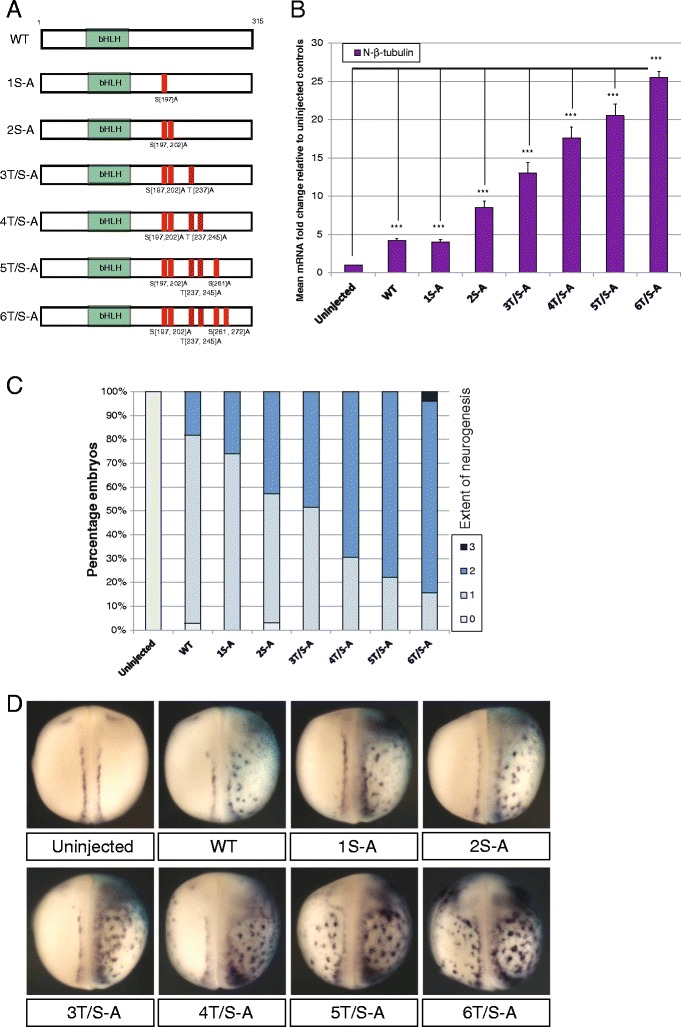
Cumulative mutation of phosphorylation sites creates step-wise increases in proneural activity (**a-d**). **a** Schematic representation of the phospho-mutant series of constructs demonstrating the SP and/or TP sites that are mutated to AP sites in each. The series consists of cumulative mutation of sites, working from N to C termini. Two-cell stage embryos were unilaterally injected with 100 pg mRNA of the respective NeuroD4 construct and assayed at stage 18 for expression of neural-β-tubulin by qRT-PCR (B[*n* = 3]), or whole embryo ISH (C[*n* = 59-77]) with representative images shown in (**d**). Views are dorso-ventral with injected side to the right; (*p* < 0.05) = *; (*p* < 0.025) = **; (*p* < 0.0125) = ***

Our data is therefore consistent with a model in which proneural activity varies with the number rather than location of sites phosphorylated. However, the data presented above do not distinguish between either a linear model, whereby mutation of each additional phosphorylation site additively increases the neurogenic activity of NeuroD4, or a threshold model, whereby mutation of a particular number of sites is enough to trigger a dramatic increase in activity. In order to investigate these two possibilities, a further panel of cumulative mutants were made, where each mutant SP/TP site was additively mutated from the N terminus to generate 1S-A, 2S-A, 3T/S-A etc. (Fig. 5a). Both qRT-PCR analysis and semi-quantitative analysis of ectopic neurogenesis as judged by in situ hybridisation show that sequential introduction of additional mutations creates a step-wise increase in neural-β-tubulin expression (Fig. 5b-d). Taken together, our data support the linear model where it is the number not the location of SP/TP sites that controls the activity of NeuroD4. Under this model, NeuroD4 may act as a “rheostat” to sense the level of proline-directed kinase activity and translate this into propensity to drive neuronal differentiation.

### Phosphorylation of NeuroD4 does not influence neuronal subtype specification

One way in which bHLH proteins can have context-dependent roles in addition to their generic proneural functions is through participation in transcription factor complexes with spatially or temporally restricted cofactors. An excellent example of this is during neuronal subtype determination, where proneural proteins form complexes with homeodomain proteins to direct subtype identity, as occurs in the spinal cord [32] or retina [33]. In some cases, this has been shown to be influenced by specific phosphorylation events [34], and in the *Xenopus* retina, subtype specification is temporally regulated by post-translational modification of bHLH factors such as NeuroD1 [35]. Mammalian Math3 has known roles in retinal subtype specification in cooperation and cross-regulation with other proneural proteins [36], and over-expression of WT NeuroD4 in the developing *Xenopus* retina promotes the formation of both ganglion and photoreceptor cells [23]. We therefore investigated whether phospho-regulation may also influence subtype specification by NeuroD4, in addition to regulating its generic proneural activity.

**Fig. 6:**
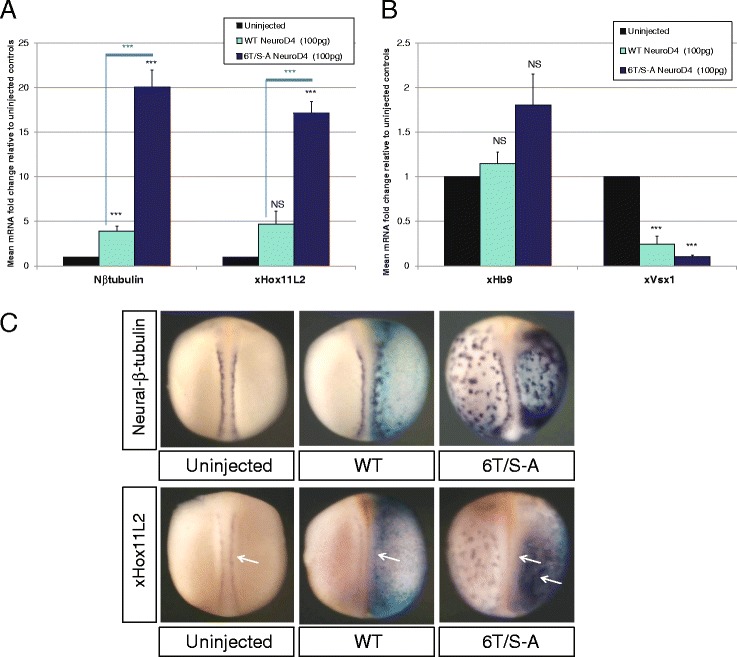
Both WT and 6T/S-A NeuroD4 generate predominantly sensory neurons (**a-e**). Embryos were unilaterally injected at the two-cell stage with 100 pg of either WT or 6T/S-A NeuroD4 mRNA and at stage 18, embryos were assayed by qRT-PCR for expression of pan neuronal gene neural-β-tubulin, sensory neuron marker xHox11L2, motor neuron marker xHb9 or interneuron marker xVsx1 (**a**, **b**). Data is shown on two separate graphs due to differences in scale [*n* = 3]. Whole embryo ISH was also conducted to compare the pattern of expression of neural-β-tubulin and xHox11L2 (**c**) [*n* = 30–38]. Views in **c** are all dorso-ventral with injected side to the right; (*p* < 0.05) = *; (*p* < 0.025) = **; (*p* < 0.0125) = ***

The bilateral stripes of *Xenopus* primary neurons give rise to sensory neurons, interneurons and motor neurons (lateral to medial), which can be distinguished by expression of xHox11L2 [37], xVsx1 [38], and xHb9 respectively [39]. All three of these marker genes are endogenously expressed at stage 18, enabling their relative expression to be determined alongside neural-β-tubulin (Fig. 6). Initial analysis of expression of subtype markers was conducted by qRT-PCR, following over-expression of WT or 6T/S-A NeuroD4 mRNA (Fig. 6a-b). The relative increase in neural-β-tubulin expression (four fold for WT and 20 fold for 6T/S-A NeuroD4) is paralleled by the increase in sensory neuron marker xHox11L2 (five-fold for WT and 17 fold for 6T/S-A NeuroD4). In contrast, no significant differences are seen in motor neuron marker xHb9, and in both WT and 6T/S-A NeuroD4 injected embryos there is a reduction in interneuron marker xVsx1. Perron and colleagues also observed a reduction of interneuron marker Pax2 following over-expression of WT NeuroD4 in non-neural ectoderm [23], so this may suggest that interneuron cell fate is being diverted towards sensory cell fate and additionally, NeuroD4-induced ectopic neurons are adopting a sensory identity. We confirmed that 6T/S-A NeuroD4 induces sensory neurons like WT NeuroD4 by in situ hybridisation (Fig. 6c). Ectopic neurons induced by over-expression of WT xNgn2 and WT NeuroD4 have previously been described as Rohon-Beard sensory neurons [23, 40] and in another vertebrate model, chick homologue NeuroM is expressed in the region of the developing sensory neurons in the dorsal root ganglia [21]. This may therefore be the default path of neuronal identity induced by this Neurogenin2-NeuroD4 transcription factor cascade, and SP/TP phosphorylation does not obviously influence neuronal subtype identity. The lack of other neuronal subtypes may be due to a lack of necessary cofactors or extrinsic signals that are required for alternative cell fates. Taken together, these results indicate that phospho-mutant NeuroD4 has considerably enhanced neurogenic activity but phospho-status has no significant effect on the subtype of neurons generated.

### NeuroD4 phospho-status affects both protein accumulation and chromatin binding

We have so far demonstrated that NeuroD4 is expressed and phosphorylated during primary neurogenesis in *Xenopus* embryos, and mutation of phosphorylation sites additively enhances the ability of NeuroD4 to drive neuronal differentiation. We then investigated the mechanisms by which 6T/S-A NeuroD4 may exert its increased neurogenic activity. We show above (Fig. 2) that phospho-mutant 6T/S-A NeuroD4 gives enhanced transcriptional activation of several downstream target genes. Two possibilities that may account for this are that phospho-mutant NeuroD4 accumulates to higher levels than the wild-type protein and/or it has a greater ability to associate with DNA. To test these, we looked at protein levels in neural plate embryos and investigated association of NeuroD4 with embryonic chromatin at this stage.

**Fig. 7:**
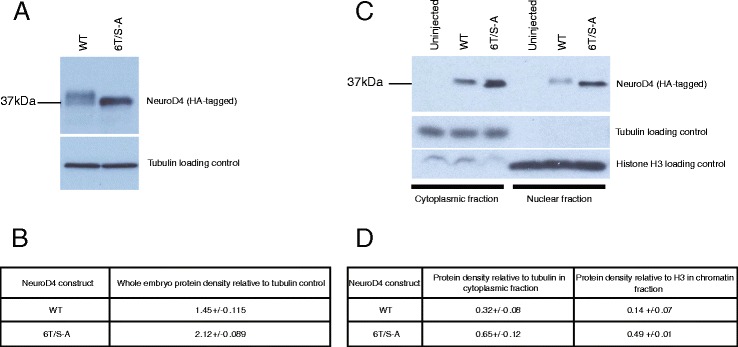
6T/S-A NeuroD4 shows enhanced protein stability and enhanced chromatin binding relative to WT NeuroD4 (**a-d**). Embryos were injected at the one cell stage with 200 pg mRNA encoding HA-tagged versions of either WT or 6T/S-A NeuroD4. **a** Western blot analysis on extracts prepared from stage 13 embryos, with tubulin as a loading control. **b** The density of the protein band for WT or 6T/S-A NeuroD4 was quantified and expressed relative to the tubulin loading control of each sample. Mean values are shown from independent duplicate samples with the standard error of the mean. **c** At stage 14, cross-linking and chromatin isolation were performed prior to western blot analysis as before. No HA-tagged NeuroD4 protein was detected in the uninjected embryos confirming antibody specificity, and no tubulin protein was detected in the chromatin fraction. **d** NeuroD4 protein bands were again quantified relative to tubulin or histone H3 bands for cytoplasmic and chromatin fractions respectively. Mean values are shown from independent duplicate samples with the standard error of the mean

In order to compare protein levels *in vivo*, HA-tagged WT or 6T/S-A NeuroD4 mRNA was injected into one cell embryos and extracts were prepared from stage 13 embryos for western blot analysis (Fig. 7a-b). WT NeuroD4 protein migrates as a broad band whereas 6T/S-A NeuroD4 migrates as a single band, demonstrating that these six SP/TP sites that are mutated in the phospho-mutant account for the phosphorylation seen in the WT protein (as discussed for Fig. 1c). The density of the NeuroD4 protein bands were calculated relative to the respective tubulin loading control: Injection of the same amount of WT or 6T/S-A NeuroD4 mRNA results in a 1.5 fold increase in 6T/S-A NeuroD4 band compared to WT NeuroD4.

We then investigated whether we also see enhanced 6T/S-A NeuroD4 associated with embryonic chromatin. After injection of equal amounts of mRNA, cross-linking was performed in stage 14 embryos and nuclei were extracted for western blot analysis. A cytoplasmic fraction was also collected and relative protein levels of WT and 6T/S-A NeuroD4 were quantified relative to tubulin loading control for the cytoplasmic fraction, and relative to Histone H3 for the chromatin fraction (Fig. 7c, d). No tubulin protein is detected in the chromatin fraction, confirming successful chromatin isolation. For the cytoplasmic fraction, we find an approximately two-fold higher protein level of phospho-mutant NeuroD4 relative to WT protein, similar to that seen in the whole embryo extracts. However, for chromatin-associated protein, we find 3.5 fold more phospho-mutant NeuroD4 compared to WT NeuroD4, demonstrating an increased DNA binding affinity. Thus, the enhanced ability of phospho-mutant NeuroD4 to drive neuronal differentiation relative to WT NeuroD4 appears to reflect a combination of a greater level of 6T/S-A NeuroD4 protein and a greater affinity for DNA compared to the WT protein.

## Discussion

### Multi-site phosphorylation is a conserved regulatory mechanism amongst proneural proteins

A sizeable number of proneural bHLH transcription factors have been identified that act at different stages in the specification, differentiation and maturation of a neuron [1], but how their different roles are controlled and co-ordinated is still poorly understood. We have previously shown that two bHLH proteins are controlled by multi-site phosphorylation on serine-proline sites; Ascl1, which regulates autonomic neuron formation in *Xenopus* [41] and generally a GABAergic phenotype (among other roles) [42], and Neurogenin2, an inducer of primary neuron formation in the developing *Xenopus* embryo [43] and generally of glutamatergic neurons [42]. Multi-site phosphorylation by cdks regulates the ability of Ascl1 and Ngn2 to control the proliferation versus differentiation decision; when cells are rapidly cycling, high cdk levels suppress the ability of these proneural proteins to drive differentiation [14–16].

NeuroD4 (otherwise known as Math3 and NeuroM) acts downstream of Ngn2 in primary neurogenesis [23] and its expression timing and pattern is consistent with a role in co-ordinating and/or driving the transition between proliferation and differentiation after that differentiation decision has been made. We show here that the ability of NeuroD4 to drive neuronal differentiation is also limited by multi-site phosphorylation. Thus, despite playing roles at differing stages of neuronal specification and differentiation, a similar role for multi-site phospho-regulation is conserved amongst bHLH proneural proteins, even though the precise kinase environment each proneural family member may be exposed to may differ. While proneural proteins can be phosphorylated by cdk1 and cdk2-dependent kinases [14, 16], other proline-directed kinases include GSK3beta, MAP kinases, cdk5 and the cdks more commonly associated with transcription including cdk7 and cdk8, may also potentially target these proteins throughout the differentiation process.

### Multi-site phosphorylation provides a rheostat-like mechanism to regulate proneural activity

Previous work investigating multi-site phospho-regulation of Ascl1 and Ngn2 indicates that the number of sites available for phosphorylation is a more important determinant of proneural protein activity than the exact location of those sites [14, 16]. We see a similar phenomenon with NeuroD4. The ability to inhibit NeuroD4-driven neurogenesis does not reside with a single SP or TP site, as no single mutant shows activity close to the full phospho-mutant; the only single residues that, when mutated, can significantly enhance NeuroD4 activity are T237 and T245. A single TP site was noted previously in Ngn2 but was not identified as playing an important regulatory role [14, 15] (and data not shown), and *Xenopus* Ascl1 has no TP sites, so NeuroD4 is the first example of TPs playing a role in proline-directed multi-site phospho-regulation of proneural protein activity.

SP and TP sites in NeuroD4 are clustered into three pairs of sites, two pairs of which are found within stretches of highly conserved residues. To see if SP/TP sites in these pairs are redundant to each other, we tested mutants where both members of each pair were mutated simultaneously. While NeuroD4 with the mutated pairs of sites show more activity than the wild-type protein or single SP/TP site mutants, each of the paired mutants results in similar enhancement of activity (approximately 1.5 to 2-fold compared to WT NeuroD4) and none show activity approaching the full phospho-mutant. Thus, mutation of single or pairs of SP/TP sites does not result in a NeuroD4 protein with the high level of activity of the full phospho-mutant. These findings support the hypothesis that it is the number of phospho-sites available and not phosphorylation at a particular location that controls proneural protein activity. To test this hypothesis further, we generated a phospho-mutant series where SP/TP sites were cumulatively mutated from the N-terminus towards the C-terminus of the protein. This cumulative phospho-mutant series can also be used to test the alternative of a threshold versus a rheostat model of phospho-regulation. Under a threshold model, mutation of a specific number of sites above a threshold would be enough to give full activation of NeuroD4. Such threshold regulation has been described for NFAT transcription factors [44]. A rheostat-like model of phospho-regulation has previously been described to control DNA binding by the ets-1 transcription factor, where mutation of additional phosphorylation sites gives an incremental increase in DNA binding [45]. In fact, using our NeuroD4 phospho-mutant series, we see a remarkably linear increase in both ectopic neurogenesis as scored by in situ hybridisation, and expression of neural-β-tubulin measured by qRT-PCR, when potential phosphorylation sites are cumulatively mutated. Taken together, the results we describe add considerable weight to a rheostat-like model whereby multi-site phosphorylation on SP and TP sites is used as a conserved mechanism to regulate the ability of proneural proteins to effect neuronal differentiation, allowing a graded response to the kinase environment.

### Under-phosphorylated proneural proteins promote neuronal differentiation through multiple mechanisms

How does phosphorylation control NeuroD4 activity? Direct downstream targets of NeuroD4 have not been systematically identified. However, xNeuroD1 and xMyT1 are clearly up-regulated by both Ngn2 and NeuroD4 in a neurogenic cascade [15, 23, 27] (Fig. 2). xNeuroD1 expression is enhanced approximately four-fold by WT NeuroD4, compared to 20-fold by WT Ngn2 (Fig. 2 and [15]) despite the injection of up to five times more NeuroD4 mRNA, demonstrating that the factors are not inter-changeable. xNeuroD1 expression is enhanced two-fold in response to phospho-mutant NeuroD4 or Ngn2 compared to the respective wild-type proteins, and similarly xMyT1 is three-fold more highly activated by 6T/S-A NeuroD4 than WT NeuroD4 (Fig. 2 and [15]), so un(der) phosphorylated Ngn2 and NeuroD4 both promote neuronal differentiation.

In contrast to Ngn2 targets that drive differentiation such as xNeuroD1 and xMyT1, we have seen that phospho-status of Ngn2 has very little effect on xDelta expression, which promotes cell non-autonomous progenitor maintenance via Notch signalling [15]. We have previously proposed that de-phosphorylation of Ngn2 results in an increase in xNeuroD1 expression with little effect on xDelta; this alone will shift the balance towards neuronal differentiation and away from progenitor maintenance. Indeed, overexpression of phospho-mutant Ngn2 leads to near total neurogenic conversion of the lateral epidermis, which overcomes any localised Notch signalling [14, 15]. In contrast to the almost uniform neurons formed on overexpression of phospho-mutant Ngn2, it is striking that ectopic neurogenesis resulting from 6T/S-A NeuroD4 occurs in distinct clumps of differentiated neurons separated by regions of epidermis (e.g. see Figs. 2, 3, 4, 5 and 6), indicative of active Notch-mediated lateral inhibition. Unlike Ngn2, 6T/S-A NeuroD4 up-regulates xDelta expression up to 3-fold compared to WT NeuroD4, which may account for the different patterns of neurogenic induction that we observe. Thus, while they are components of the same unidirectional cascade, Ngn2 and NeuroD4 show distinct properties in both regulation of neuronal differentiation and maintenance of lateral inhibition.

Cdks can phosphorylate proneural proteins on SP/TP sites [14, 16]. Our model predicts that as the cell cycle lengthens and cdk activity drops, de-phosphorylation of proneural proteins increases and drives neuronal differentiation further [14–16]. But what causes the initial drop in cdk levels that potentiates this drive to differentiate? Cdk inhibitors of the Cip/Kip family play an important role in both inhibiting cdks and also directly potentiating neuronal differentiation [28, 46]. While proneural protein expression results in an increase in cdki protein levels in P19 cells [47], these cdkis are generally not shown to be direct transcriptional targets of proneural proteins [13, 29]. We see that the single cdki that is required for primary neurogenesis in *Xenopus*, p27Xic1 [28] is not transcriptionally up-regulated by WT NeuroD4. However, two-fold up-regulation of p27Xic1 is seen with 6T/S-A NeuroD4 (Fig. 2). Up-regulation of cdkis by un(der)phosphorylated proneural proteins offers a feedback mechanism to further drive neuronal differentiation once it has been initiated; enhancing the expression of cdkis will both inhibit cdk activity and stabilise proneural proteins and so potentiate neuronal differentiation. However, what brings about an initial reduction in proline-directed kinase activity, which results in accumulation of un(der)phosphorylated proneural proteins and so enhanced proneural protein activity, remains unclear.

We have previously shown than un(der)phosphorylated Ngn2 shows enhanced DNA binding compared to its phosphorylated counterpart [14]. Western blot analysis from embryos injected with equal amounts of either WT or 6T/S-A NeuroD4 mRNA reveals 1.5 times more phospho-mutant NeuroD4 protein compared to WT protein. Our previous work has shown that phospho-status of Ngn2 does not affect its intrinsic protein stability *in vitro* [19], but 9S-A Ngn2 shows differentially enhanced stability relative to WT Ngn2 when co-incubated with E proteins [15]. Our data suggest that NeuroD4 may show similar preferential stability of the phospho-mutant form in the endogenous environment of the embryo where E proteins are abundant [48]. As well as this moderately enhanced overall abundance, we found that 3.5 fold more 6T/S-A NeuroD4 was associated with chromatin *in vivo* compared to the wild-type protein. This reinforces our findings *in vitro* and in tissue culture cells showing greater association of phospho-mutant proneural proteins with E box-containing DNA [14].

Together with our previous work on Ngn2 and Ascl1 phospho-regulation, the results presented above strengthen and widens our model whereby un(der)phosphorylation of multiple proneural transcription factors enhances promoter dwell-time on their downstream target promoters. Enhanced dwell-time disproportionately promotes activation of targets that require extensive chromatin modification for transcription to occur, particularly genes associated with differentiation such as xNeuroD1 and xMyT1; proneural proteins can associate with chromatin modification enzymes such as histone acetyltransferases and swi/snf-family nucleosome remodellers, and underphosphorylated proneural proteins will recruit them more effectively to target promoters [24, 49]. Conversely, multi-site phosphorylation of proneural proteins found when proline-directed kinases such as cdks are high, limits their chromatin binding and favours progenitor maintenance. Thus, post-translational modification of proneural proteins active at sequential stages in primary neurogenesis can be used to sense the kinase environment and use this to regulate neuronal differentiation.

## Conclusions

Here we show that proneural bHLH protein NeuroD4 is expressed and phosphorylated during primary neurogenesis in *Xenopus* embryos. Homologues of NeuroD4 in other species show strong sequence conservation of potential proline-directed kinase sites (SP/TP). We have used the *Xenopus* embryo model to demonstrate that NeuroD4 proneural activity is regulated by phosphorylation on up to six SP/TP sites. A multiply phospho-mutant version of NeuroD4 is able to drive significantly enhanced neurogenesis compared to the wild-type protein, although the subtype of neurons generated is not altered. Extensive mutational analysis demonstrates that it is the number rather than location of available phospho-sites that is the critical factor for regulation of NeuroD4 activity. NeuroD4 dephosphorylation promotes neuronal differentiation through a combination of increased protein stability, increased chromatin binding, and consequent up-regulation of a range of target genes that also promote differentiation. Taken together with previous findings about regulation of the proneural proteins Ngn2 and Ascl1, we propose that a conserved rheostat-like mechanism of phospho-regulation enables proneural protein activity to control the balance between progenitor maintenance and differentiation in response to the cellular kinase environment.

## Methods

### Animal care

*Xenopus laevis* were housed, bred and treated according to the guidelines approved by the UK Home Office under the Animal (Scientific Procedures) Act 1986. All animal work has been carried out under UK Home Office Licence and has passed an Institutional ethical review committee assessment, undertaken by the Animal Welfare and Ethical Review Committee (AWERC) at the University of Cambridge.

### Plasmids and constructs

*X. laevis* wild-type (WT) *NeuroD4* in pCS2+ (Genbank accession number NM 001087744) was a kind gift from Professor Shin-Ichi Ohnuna (University College London, UK). For western blot detection, HA-tagged WT *NeuroD4* in pCS2+ was generated by PCR. Primers 5′-GATCGAATCCACCATGTCAGAGATGGTCAATGTG-3′ and 5′- GATCCTCGAGTTAAGCGTAATCTGGAACATCGTATGGGTATTCAGTGAATATAGCATGGTGTG-3′ were used to amplify the coding region of *NeuroD4*, adding a single HA tag at the 3′ end before the stop codon, and introducing EcoR1 and Xho1 restriction enzyme sites to the extreme 5′ and 3′ ends respectively. This PCR product was digested with EcoR1 and Xho1 and ligated into a blank pCS2+ vector. All phospho-mutant constructs, including the HA-tagged 6T/S-A *NeuroD4*, were subsequently generated from either the untagged WT *NeuroD4* or HA-tagged WT *NeuroD4* constructs by site directed mutagenesis. Single or multiple site mutation were performed using the QuikChange II Site-Directed Mutagenesis Kit (Agilent Technologies) or the QuikChange Multi Site-Directed Mutagenesis Kit (Agilent Technologies) respectively, each according to manufacturer’s instructions and using the PCR primers as shown in Table 1. All sequences were confirmed by Sanger sequencing of plasmid DNA. Nucleotide and protein sequence alignments were conducted using ClustalW software [50].

**Table 1:** Primer sequences for site-directed mutagenesis of NeuroD4

Mutation	Sense primer 5′ to 3′	Anti-sense primer 5′ to 3′
Xath_S197A	CATACTTATAATTACCAGGCCCCAGGACTACCCAG	CTGGGTAGTCCTGGGGCCTGGTAATTATAAGTATG
Xath_S202A	GGCCCCAGGACTACCCGCTCCTCCTTATGGTAAC	GTTACCATAAGGAGGAGCGGGTAGTCCTGGGGCC
Xath_T237A	CATACACTTAACTGTACCGCTCCACCATATGAAGGAG	CTCCTTCATATGGTGGAGCGGTACAGTTAAGTGTATG
Xath_T245A	TCCACCATATGAAGGAGCTCTAGCACCTCCACTCA	TGAGTGGAGGTGCTAGAGCTCCTTCATATGGTGGA
Xath_S261A	GGTAATTTTTCTTTGAAGCAAGATAGTGCACCCGATATGGATAAAT	ATTTATCCATATCGGGTGCACTATCTTGCTTCAAAGAAAAATTACC
Xath_S272A	AATCATATGCATTCAGGGCCCCCTATCCAGCTCTT	AAGAGCTGGATAGGGGGCCCTGAATGCATATGATT

### *Xenopus laevis* embryo manipulation

*X. laevis* eggs were obtained by standard hormone methods of induction, and subsequently fertilised *in vitro*. pCS2+ plasmids containing the various *NeuroD4* constructs were linearised with Not1 enzyme and capped mRNA was transcribed *in vitro* using the SP6 mMessage mMachine® kit (Ambion). Unless otherwise stated, two-cell-stage embryos were unilaterally injected into the animal pole with 100 pg NeuroD4 mRNA in a total volume of 10 nl, with co-injection of 500 pg GFP and β-gal mRNA to enable lineage tracing. Embryos were subsequently cultured at 16 °C in Ficoll solution (4 % w/v Ficoll, 0.2x MBS, 50ug/ml Gentamycin in water) and staged according to the methods of Niewkoop and Faber [51]. At stage 18, embryos were either snap-frozen for qRT-PCR analysis (see below), or fixed in MEMFA (4 % formaldehyde, 100 mM MOPS, 2 mM EGTA, 1 mM MgSO_4_, pH 7.5) for 90 min, washed twice in phosphate-buffered saline (PBS), followed by PBS supplemented with 2 mM MgCl_2_, and embryos were stained with 1 mg/ml X-gal (5-bromo-4-chloro-3-indolyl-beta-D-galactopyranoside) in X-gal mixer (5.35 mM K_3_Fe(CN)_6_, 5.35 mM K_4_Fe(CN)_6_, 1.2 mM MgCl_2_, 0.1 % sodium deoxycholate, 0.2 % NP-40 in PBS). Embryos were again washed twice in PBS and dehydrated in methanol prior to storage at −20 °C.

### Whole mount in situ hybridisation

Dig-oxigenin-labelled anti-sense probes were synthesised from the following plasmids: *X. laevis neural-β-tubulin* [52]; *X. laevis p27Xic1* [52]; *X. laevis xMyt1* [27], *X. laevis xNeuroD1* (a kind gift from Professor Shin-Ichi Ohnuna (University College London, UK); linearised with BamH1 and transcribed from T7) and *X. laevis xHox11L2* [23]. Whole mount ISH was performed using a BioLane™ HTI in situ robot (Holle and Huttner) with washes and composition of solutions as described in [53], with some modifications. RNase treatment was omitted and blocking was performed using 2 % Blocking Reagent (Roche) and 20 % heat inactivated lamb serum in maleic acid buffer (0.1 M maleic acid, 0.15 M NaCl, pH 7.5). This was then followed by incubation in the same solution containing 1:5000 anti-digoxigenin antibody-coupled to alkaline phosphatase (Roche). The chromogenic reaction was conducted in BM purple (Roche) and terminated by methanol washes prior to overnight fixation in MEMFA. Embryos were then washed in 0.1 % Tween-20 in PBS, and bleached on a light box for 1 h in bleaching solution (10 % H_2_O_2_, 5 % formamide, 0.1 % SSC), prior to PBS washes and storage in MEMFA at room temperature. Embryos were scored for neurogenesis as described in Additional file 1 and representative embryos from each category were photographed using a CoolSNAP colour camera (RS Photometrics) and Openlab™ software.

### Quantitative real-time PCR (qRT-PCR)

Embryos for qRT-PCR analysis were co-injected with 500 pg mRNA encoding GFP, and GFP expression was subsequently used to confirm successful injection. Samples of four embryos were snap-frozen at stage 18 and whole embryo RNA was extracted using the Qiagen RNeasy® Mini Kit. Template cDNAs were synthesised using the QuantiTect® Reverse Transcription Kit (Qiagen), and qRT-PCR was performed using the Quantifast® SYBR Green PCR Kit (Qiagen) in a LightCycler® 480 (Roche). Thermal cycling conditions: 95 °C for 5 min, then 45 cycles of 95 °C for 10 s, 60 °C for 10 s and 72 °C for 20 s. Primer sequences were as shown in Table 2.

**Table 2:** Primer sequences used in quantitative real-time PCR

Name	Sequence (5′ to 3′)	GenBank accession number of gene
xEF1α F	CACCATGAAGCCCTTACTGAG	NM 001087442
xEF1α R	TGATAACCTGTGCGGTAAATG	
N-β-tubulin F	TGGATTTGGAACCAGGCA	NM 001086064
N-β-tubulin R	GCTCAGCTCCTTCGGTGTA	
p27Xic1 F	ACTGAAGGAAATCCAAGCGTC	NM 001114803
p27Xic1 R	CTGGCTGTAGAAACTGGGCAT	
xDelta F	GCCCCAGAGATGATGCTTTC	BC 070634
xDelta R	GCCTTGCCAACCCACTCTACATT	
xMyt1 F	TAAAGTCGGGTCAAGCGGAA	NM 001088192
xMyt1 R	TTCATCTCTGGGTTCAGTGCC	
xNeuroD1 F	GACTCAACGATGCCCTGGA	NM 001085794
xNeuroD1 R	CCCGCTACTAGATTGGTGGTG	
xHb9 F	TCCGAGCACACTGACAGC	NM 001096823
xHb9 R	CATCGGGTGTCCATACAGG	
xHox11L2 F	CCCAGGACTTCATTCACCA	NM 001085747
xHox11L2 R	GGTTTTGACTTGGGCATCTG	
xVsx1 F	TTTGCCATCACTGACCTGCTT	NM 001096722
xVsx1 R	AAGCCACACAGAAAGCCCAG	

### Western blotting

For detection of NeuroD4 phosphorylation, embryos were injected bilaterally at the two-cell stage with 300 pg WT NeuroD4-HA mRNA. For comparison of WT and 6T/S-A NeuroD4 protein stability, embryos were injected at the one cell stage with 200 pg of the respective HA-tagged mRNA. Samples of five embryos were snap-frozen on dry ice at stage 13 and subsequently lysed in 50ul of pre-chilled IP-buffer (50 mM Tris pH7.9, 100 mM NaCl, 5 mM EDTA, 0.1 % Triton-X-100, 50 mM β-glycerophosphate, with 1:25 protease inhibitor cocktail (Roche)). Lysed embryos were centrifuged at 4 °C for 5 min at 16 000 xg and the supernatant re-spun and final supernatant used as the protein extract. Protein was quantified in the extract using a BCA assay (Thermo Scientific) according to manufacturer’s instructions, and 30ug was subsequently used for Western blot analysis in a total volume of 10 ul. For detection of NeuroD4 phospho-status, samples were incubated at 30 °C for 1 h in the presence or absence of Lambda protein phosphatase (NEB). 3.5 ul of SDS-loading buffer containing reducing agent was then added to each sample and incubated at 70 °C for 10 min prior to brief 15 s centrifugation. Samples were loaded on to a pre-cast BioRad Criterion™ TGX™ 18 % gel using the BioRad precision plus protein dual colour standard as a ladder. The gel was run for 2 h at 200 mV in running buffer (25 mM Tris, 192 mM glycine, 0.1 % SDS, pH8.3). Transfer was conducted at 100 mV for 45 min at 4 °C, using pre-chilled transfer buffer (6 g Trizma Base, 28.8 g Glycine, 400 ml Methanol in total 2 L volume) and PVDF membrane activated in methanol. Blocking was performed for over 1 h in 4 % milk in TBS with 0.1 % tween and the membrane was cut at just below the 50KDa level to enable separate antibody incubation to detect either the NeuroD4 protein or Tubulin loading control. Primary antibodies were used at 1:2000 dilution in 2 % milk in TBS-T and applied for at least 1 h at room temperature or 4 °C overnight: rat HRP-conjugated anti-HA (clone 3 F10) antibody (Roche) and mouse anti-α-tubulin antibody (Sigma). Membranes were washed at least 5 times in TBS-T and anti-tubulin antibody was detected using an HRP-conjugated anti-mouse antibody at 1:10000 dilution in 2 % milk in TBS-T for 1 h at room temperature. Membranes were washed again at least 5 times prior to Amersham ECL detection reagent reaction (GE Healthcare) according to manufacturer’s instructions. Experiments were conducted in independent duplicate and representative images are shown. For protein quantification in Fig. 7, ImageJ software was used to calculate the density of the NeuroD4 protein band relative to the density of the tubulin loading control band. An average value was calculated for independent repeats and error margins were calculated using the standard error of the mean.

### Assay of chromatin association

Embryos were injected at the one cell stage with 200 pg HA-tagged mRNA as before. At stage 14, embryos were transfered to glass vials and rinsed 3 times with distilled water. Embryos were then incubated at room temperature for 30 min in 1 % formaldehyde in 0.1 % MBS solution, prior to quenching in 0.25x MBS containing 125 mM glycine for 30 min. Embryos were then washed twice for 30 min total in 0.25x MBS, and 30 embryos per condition were snap-frozen on dry ice. Nuclei were extracted using a modified protocol for chromatin isolation. Embryos were homogenised in 50ul of Buffer E1 (50 mM Hepes-KOH pH7.5, 140 mM NaCl, 1 mM EDTA pH8.0, 10 % Glycerol, 0.5 % NP-40, 0.25 % TritonX-100, 1 mM DTT, 0.2 mM PMSF and protease inhibitor cocktail (Roche)) and spun for 2 min at 3500 rpm at 4 °C. The supernatent was collected and retained as the cytoplasmic fraction, and 10 ul of this was subsequently used for Western Blot. The lipid residue was removed and the remaining pellet was resuspended in 1 ml of Buffer E1, followed by a repeat centrifugation step. The pellet was again resuspended in 1 ml of Buffer E1, and samples were rested on ice for 10 min prior to centrifugation. The resulting pellet was then resuspended in 50ul Buffer E2 (10 mM Tris pH8.0, 200 mM NaCl, 1 mM EDTA pH8.0, 0.5 mM EGTA pH 8.0, 0.2 mM PMSF and protease inhibitor cocktail (Roche)), and the process was repeated with 2 cycles of centrifugation and resuspension, followed by 10 min rest on ice before final centrifugation. The resulting pellet was resuspended in 250ul of Buffer E3 (500 mM Tris pH8.0, 500 mM NaCl and protease inhibitor cocktain (Roche)), and this represents the chromatin fraction. 20ul of the chromatin fraction was then DNAse treated (Qiagen) at room temperature for 15 min and Western Blot analysis was conducted as described above using 10ul samples. A 4-20 % gel was used and antibodies were as above, with the additional use of Anti-Histone H3 antibody (Abcam) and anti-rabbit secondary antibody (Sigma). ImageJ software was again used to quantify protein bands and the density of WT or 6T/S-A NeuroD4 protein was calculated relative to tubulin protein or histone H3 protein for cytoplasmic and chromatin fractions respectively.

### Statistical analysis

For qRT-PCR data, mRNA expression was normalised to expression of the housekeeping gene *Elongation Factor 1α (EF1α)*, and for analysis, mRNA levels in the injected embryos were calculated relative to stage-matched uninjected control embryos. Mean values and the standard error of the mean (s.e.m.) were calculated from at least three independent experiments (*n* = 3). Statistical significance was determined using a paired two-tailed Student’s *t*-test with (*p* < 0.05) = *; (*p* < 0.025) = **; (*p* < 0.0125) = ***. For in situ hybridisation data, embryos were scored for the extent and pattern of marker gene expression on the injected side of the embryo relative to the uninjected side and uninjected control embryos. Additional file 1 provides representative images and descriptions for the scoring of neural-β-tubulin expression, and similar principles were applied to scoring the expression of p27Xic1, xMyt1 and xNeuroD1. Experiments were conducted in independent duplicate or triplicate and the n numbers reported refer to the range of total numbers of embryos in each injection category. Statistical significance was determined between categories using a Fisher’s Exact Test for Count Data, and *p* values are illustrated as described above.

## Additional file

Additional file 1:
**Representative embryo images to demonstrate** **semi-quantitative** **scoring system used for in situ hybridisation data.** Neurogenesis was graded by comparing the extent and pattern of neural β-tubulin expression following in situ hybridisation on the injected side of the embryo relative to the uninjected side and uninjected control embryos. Scores were assigned as: 0, no difference; +1, mild increase in neurogenesis within the neural plate, with or without occasional ectopic neurons on the injected side; +2, moderate increase in neurogenesis with ectopic expression of neural β-tubulin occurring in patches on the injected side and sometimes bilaterally; +3, marked increase in neurogenesis with extensive ectopic expression of neural β-tubulin in a more homogenous pattern on the injected side and sometimes bilaterally.

## Abbreviations

bHLH: Basic helix-loop-helix
cdk: Cyclin dependent kinases
cdki: Cyclin dependent kinase inhibitor
ISH: In situ hybridisation
Ngn2: Neurogenin2
PBS: Phosphate-buffered saline
SP: Serine-proline
TP: Threonine-proline
VZ: Ventricular zone
WT: Wild-type
