# Supplementary figures and images for: Multi-site phosphorylation regulates NeuroD4 activity during primary neurogenesis: a conserved mechanism amongst proneural proteins
Source: Neural Dev. 2015 Jun 18;10:15. doi: 10.1186/s13064-015-0044-8 (PMC4494719; doi:10.1186/s13064-015-0044-8)

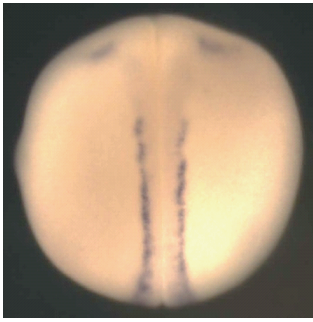

Grade 0

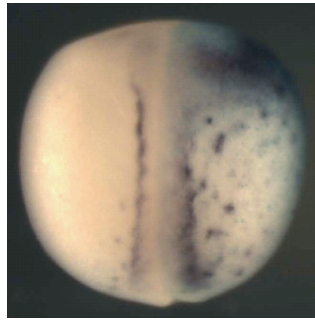

Grade 1

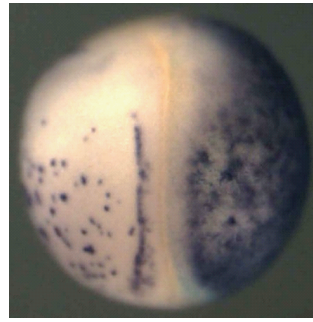

Grade 2

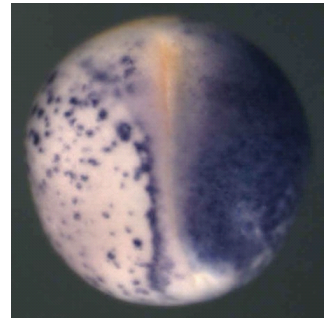

Grade 3

Supplement: Additional file 1: — Representative embryo images to demonstrate semi-quantitative scoring system used for in situ hybridisation data. Neurogenesis was graded by comparing the extent and pattern of neural β-tubulin expression following in situ hybridisation on the injected side of the embryo relative to the uninjected side and uninjected control embryos. Scores were assigned as: 0, no difference; +1, mild increase in neurogenesis within the neural plate, with or without occasional ectopic neurons on the injected side; +2, moderate increase in neurogenesis with ectopic expression of neural β-tubulin occurring in patches on the injected side and sometimes bilaterally; +3, marked increase in neurogenesis with extensive ectopic expression of neural β-tubulin in a more homogenous pattern on the injected side and sometimes bilaterally. [file 13064_2015_44_MOESM1_ESM.pdf]
